# The Current Situation of Anaesthesia for Hysteroscopy in Mainland China: A National Survey

**DOI:** 10.3390/jpm13101436

**Published:** 2023-09-26

**Authors:** Lili Huang, Qing Yu, Ziyu Zhu, Ping Huang, Xibing Ding, Xiaowen Ma, Yuwen Chen, Diansan Su

**Affiliations:** Department of Anesthesiology, Renji Hospital, School of Medicine, Shanghai Jiaotong University, Shanghai 200127, China; xinxiangyiban5@163.com (L.H.); jeanyuqing@outlook.com (Q.Y.); zhuziyu@renji.com (Z.Z.); apple_h_p@aliyun.com (P.H.); dingxibing@renji.com (X.D.); qiji8628@163.com (X.M.); chenyuwen@renji.com (Y.C.)

**Keywords:** China, hysteroscopy, anaesthesia, sedation, monitor, personnel

## Abstract

**Purpose:** The need for anaesthesia or analgesia for performing hysteroscopy remains debatable. This study aimed to conduct an overview of the situation of anaesthesia for hysteroscopy in mainland China. **Methods:** Two questionnaires were separately designed for anaesthesiologists and gynaecologists and distributed to every medical institution that performed hysteroscopic procedures on patients with infertility in mainland China. Electronic questionnaires were distributed via WeChat, and data on anaesthesia regimen, monitoring parameters, procedure number, and other information were collected. **Results:** Reproductive technology is conducted by 536 institutions in mainland China. The survey received 491 responses from anaesthetists (91.6%) and 436 from gynaecologists (81.3%). In 2021, 552,225 hysteroscopies were conducted in 268 medical centres. The average percentage of hysteroscopy under anaesthesia is 63.8% in 2021, wherein 47.3% of institutions have an anaesthesia percentage of >75%. Propofol and opioid analgesics, such as fentanyl and sufentanil, were the most commonly used intravenous anaesthetics. All sedations were performed by anaesthesiologists. Monitoring parameters included pulse oxygen saturation (98.9%), electrocardiogram (91.6%) and noninvasive blood pressure (91.3%). An anaesthesiologist-to-operating room ratio of <1 was observed in 31.3% of medical institutions. Surprisingly, 52.4% of medical institutions performing hysteroscopy had no postanaesthesia care unit (PACU). Most institutions with PACU were equipped with independent oxygen sources, suction and monitors. Both rigid and flexible hysteroscopes (rigid hysteroscope, 45.1%; flexible hysteroscope, 4.5%; both types, 50.4%) were used, and the hysteroscopic diameter was ≤5 mm in 60.3% of medical centres. **Conclusions:** China performs a large number of hysteroscopies, and sedation is the most frequently used anesthesia regimen. However, issues such as inadequate emergency support devices, insufficient personnel and weak resuscitation management after anaesthesia, have been observed.

## 1. Introduction

The morbidity of infertility has increased in recent years. The ‘Core Information on Infertility Prevention and Health Education’ published by the National Health Commission of the people’s republic of China in October 2021 reported a 7–10% morbidity of infertility in China [1]. Consistently, infertility is estimated to affect 8–12% of reproductive-aged couples worldwide [2]. Intrauterine pathology, such as endometrial polyps, fibroids, septa or intrauterine adhesions play a role in female infertility [2,3]. Hysteroscopy has been regarded as the gold standard for the treatment of intrauterine pathology and is most frequently used in the workup of patients with infertility having suspected cavity abnormalities [4,5,6]. It can reveal the intrauterine abnormalities that may have been missed by transvaginal ultrasound [7,8]. Hysteroscopic procedures are increasingly performed in an outpatient, office-based setting due to hysteroscopic technique advancements. The need for anaesthesia or analgesia for performing hysteroscopy remains debatable. The most important limiting factor of performing hysteroscopic treatments is patient discomfort, although few studies indicated that hysteroscopy was well tolerated in some females and could be performed without anaesthesia [9,10,11]. Pain is one of the primary reasons for failed hysteroscopy procedures [12]. Various anaesthesia and pain-relief methods have been described, including general anaesthesia, sedation, local anaesthesia, oral analgesics, etc.

Reportedly, experienced hysteroscopists prefer local anaesthesia in the office operating room setting, while some choose to perform hysteroscopies under general anaesthesia, believing it to be a quieter and more efficient approach [13]. Procedural sedation was a good alternative for patients with anxiety and expected painful procedures. Lisa and colleagues demonstrated the safety and feasibility of performing therapeutic hysteroscopies under procedural sedation in an outpatient setting, with low pain scores and a high level of patient satisfaction [14].

The status of hysteroscopy in China, especially in aspects of anaesthesia, remained unclear, although studies reported that hysteroscopy was a well tolerated procedure and many females found it acceptable [9]. The answers to the following questions would help build an overview of hysteroscopy in China and find the potential questions and help improve the quality of sedation for hysteroscopy in China: Was sedation administrated with hysteroscopy? Who performed the sedation? What kind of sedation and how many cases of hysteroscopy were performed per year?

## 2. Methods

### 2.1. Questionnaire Design

This survey was approved by the Institutional Review Board of Renji Hospital affiliated to Shanghai Jiaotong University School of Medicine. A pilot survey was conducted from 1st to 26th May 2022. The final two WeChat-based electronic questionnaires were separately designed for anaesthesiologists and gynaecologists, including 20 and 9 questions, respectively, after discussion with experts in the ethics community. The questionnaires were presented in Chinese and composed of the beginning time and numbers of hysteroscopies in recent years, anaesthesia, monitoring parameters and devices, the situation of postanaesthesia care unit (PACU), lens type and diameter (Supplementary Materials S1 and S2). A cover letter and informed consent were attached to the beginning of each questionnaire to clarify the purpose and intentions of the study and to inform the participants that the survey would collect their surnames, institution names and telephone numbers to validate data. Participants were notified that their responses and privacy would be maintained in password-protected computers and that any identifying information would be delinked after analysis and publication. Survey questions could be answered only after respondents agreed with these terms.

### 2.2. Conduct of the Survey

The study was conducted from 7th June to 31st August 2022, including questionnaire distribution and collection, data rechecking, verification and cleaning. Questionnaires were sent to the anaesthesiologists and gynaecologists who were the lead gynecological anaesthetist and gynaecologists in charge of hysteroscopy at 536 assisted reproductive centres that were approved for conducting reproductive hysteroscopic procedures through the most prevalent chatting application, WeChat (Tencent, Inc., Shenzhen, China), as previously described [15]. Briefly, nearly everyone has a WeChat identity and people can chat, work and transfer files through WeChat in China. Therefore, we uploaded the questionnaires to the WeChat group of the Chinese Anaesthesiologist Association, and each member in this group shared this questionnaire with their respective local WeChat anaesthesiologist association groups and gynaecologists’ groups. Questionnaires were powered by WJX (Changsha Ranxing IT Ltd., Changsha, China), and respondents could click a link to answer questions. The data from the questionnaires could be exported in Excel format. Data cleaning was conducted after collecting questionnaires to the greatest extent possible. We contacted the respondents to recheck and verify questionable items.

### 2.3. Statistical Analysis

Data analysis was conducted from September to October 2022. Means, standard deviations, and 95% confidence intervals were reported for symmetric distributed continuous variables. Median, interquartile range (IQR), minimum and maximum were calculated for nonsymmetric continuous variables. Categorical data were presented as frequency and percentage. All data were collected with Microsoft Access and analysed by Prism 7.0 (GraphPad Software, La Jolla, CA, USA).

## 3. Results

### 3.1. Institutions Carried out Assisted Reproductive Technology and Numbers of Hysteroscopy Increased Rapidly in China

**Figure 1:**
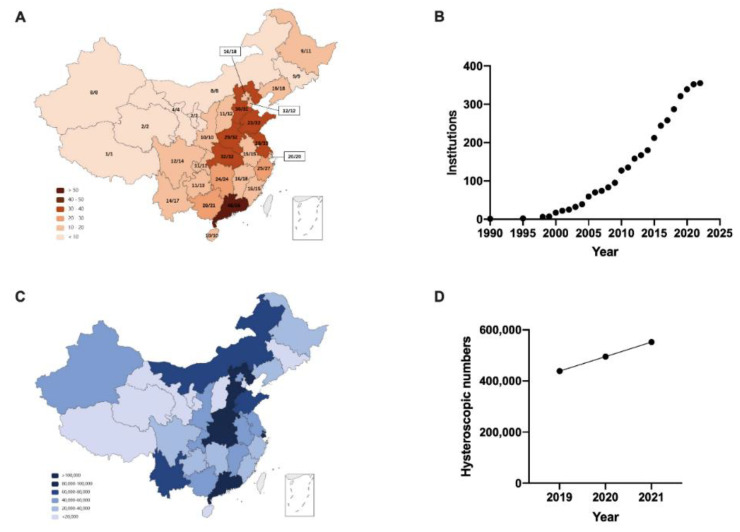
National distribution of the medical institutions and numbers of hysteroscopies in China mainland. (**A**) Map of 536 institutions that approved to carry out human assisted reproductive technology in 32 provinces, autonomous regions and municipalities. The ratio of the institutions that replied to total institutions is presented by n/n in each province. (**B**) Responses to the question: in which year did your institution carry out the first hysteroscopy? The dots presented the cumulative numbers of insitutions carried out hysteroscopy in each year. (**C**) Map of cumulative quantity of hysteroscopy in 2019–2021. (**D**) Numbers of hysteroscopy in 268 institutions that replied in 2019–2021.

In mainland China, 536 medical institutions are qualified for assisted reproductive technology, showing the characteristics of regional distribution (http://www.nhc.gov.cn/cms-search/xxgk/getManuscriptXxgk.htm?id=0cf528f318f84eafaf19b6e18ac2c44f (accessed on 7 February 2022)). Guangdong Province has the largest number with 56 institutions, followed by Jiangsu Province with 33 institutions, Shandong Province with 32 institutions, Hubei Province with 32 institutions and Hebei Province with 31 institutions. The Tibet Autonomous Region is the least, with only one, and Ningxia Hui Autonomous Region and Qinghai Province have two institutions each (Figure 1A). We received 491 responses from anaesthesiologists (491/536, response rate 91.6%) and 436 responses from gynaecologists (436/536, response rate 81.3%). The results revealed that 355 medical institutions conducted hysteroscopic procedures, accounting for 72.3% (355/491). Hysteroscopy was first conducted in China in the 1990s and developed rapidly in the past 10 years from 2010 to 2020 (Figure 1B). The statistical results showed an increasing number of hysteroscopic operations year by year from 2019 to 2021, of which the total number of operations nationwide was approximately 435,248 in 2019, 495,396 in 2020 and 552,225 in 2021 (Figure 1C,D). According to the collected information in our survey, the ratio of operative hysteroscopy was approximately 33.9% in 2021.

### 3.2. Sedation Was the Most Common Anesthetic Management Method Used in Hysteroscopy

**Figure 2:**
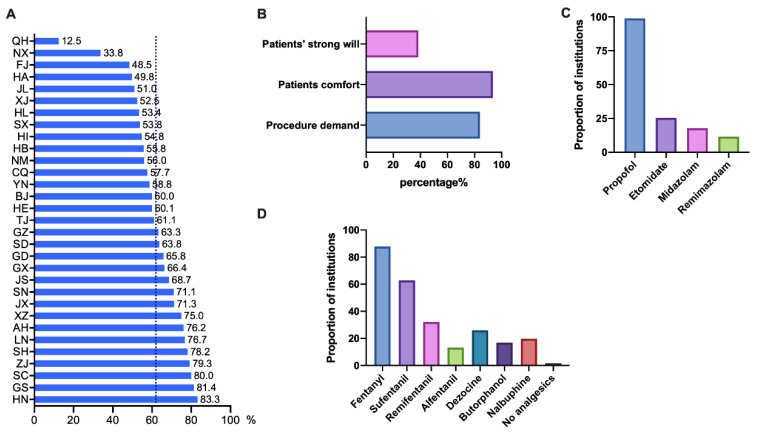
The proportion and reasons of hysteroscopy anesthesia, and the most frequently used anesthetics for sedation. Responses to the questions: (**A**) What was the proportion of anesthesia applied in hysteroscopic procedure in your reproductive institution in 2021? (**B**) What were the reasons for the high proportion of anesthesia in your institution? (**C**) What were the most commonly used sedation anesthetics during sedation of hysteroscopy? (**D**) What were the most commonly used analgesics? Abbreviations: AH, Anhui; BJ, Beijing; CQ, Chongqing; EtCO2, end-tidal carbon dioxide; FJ, Fujian; GD, Guangdong; GS, Gansu; GX, Guangxi; GZ, Guizhou; HA, Henan; HB, Hubei; HE, Hebei; HI, Hainan; HL, Heilongjiang; HN, Hunan; JL, Jilin; JS, Jiangsu; JX, Jiangxi; LN, Liaoning; NM, Inner Mongolia; NX, Ningxia; QH, Qinghai; SC, Sichuan; SD, Shandong; SH, Shanghai; SN, Shaanxi; SpO2, pulse oxygen saturation; SX, Shanxi; TJ, Tianjin; XJ, Sinkiang; XZ, Tibet; YN, Yunnan; ZJ, Zhejiang.

We investigated the situation of anaesthesia methods and proportions and anaesthetics used in sedation to understand whether sedation was administrated when performing hysteroscopy. In 2021, the national average proportion of anaesthesia for hysteroscopy was 61.9%, and 47.3% of institutions had >75%. Hunan Province had the highest proportion of anaesthesia, which was 83.3%, while Qinghai Province was the lowest, at only 12.5% (Figure 2A). The reasons for the high proportion of anaesthesia concluded to make patients comfortable (93.6%), operation requirements (83.9%) and patient’s strong willingness (38.5%) (Figure 2B). 

Sedation accounted for >75% in 44.2% of institutions, and general anaesthesia with laryngeal mask airway accounted for >75% in 13% of hospitals. Few institutions chose general anaesthesia with endotracheal tube intubation, intraspinal anaesthesia and local anaesthesia (Table 1).

Anaesthetics used in sedation varied from hospital to hospital, but mainly were sedatives plus opioid analgesics. Almost all anaesthesiologists chose propofol as the anaesthetic sedative (98.9%). The main analgesics included fentanyl (87.9%), sufentanil (62.8%) and remifentanil (32.1%) (Figure 2C,D).

### 3.3. Regular Nasal Catheter Was the Most Widely Used Oxygen Delivery Device and Monitoring Indicators Were Similar during Sedation of Hysteroscopy

**Figure 3:**
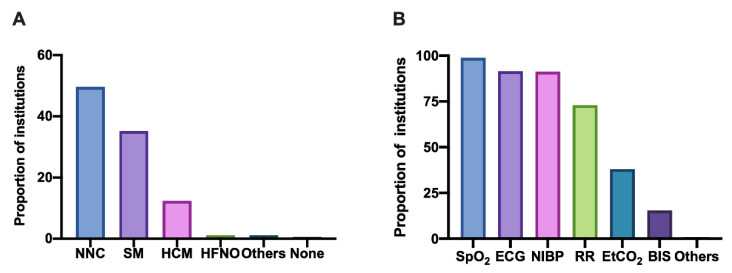
The oxygen delivery technique and monitoring parameters applied in hysteroscopy. Responses to the questions: (**A**) Which oxygen delivery technique was applied in sedation of hysteroscopy? (**B**) Which monitoring parameters were used during sedation of hysteroscopy? Abbreviations: NNC, normal nasal catheter; SM, standard mask; HCM, high concentration mask; HFNO, high flow nasal oxygen; SPO_2_, pulse oxygen saturation; ECG, electrocardiography; NIBP, noninvasive blood pressure; RR, respiratory rate; ETCO_2_, end-tidal carbon dioxide; BIS, bispectral index.

Different ways of oxygen administration are used in sedation for hysteroscopy between institutions. Regular nasal catheter oxygen (1~6 L/min) was the most frequently used method (49.6%). Approximately 35.2% of anaesthesiologists selected the standard mask for oxygen supply; however, only 1.1% chose high-flow nasal oxygen (HFNO) (>30 L/min), which meant poor HFNO application in sedation for hysteroscopy (Figure 3A).

Almost all institutions monitored pulse oxygen saturation (SPO_2_) (98.9%), electrocardiography (ECG) (91.6%) and noninvasive blood pressure (NIBP) (91.3%) during the hysteroscopic procedures. Respiratory frequency was monitored by 73.0% of institutions, and the end-tidal carbon dioxide (ETCO_2_) was monitored less frequently (38.0%) (Figure 3B).

### 3.4. The Rescue Facilities and Personnel Were Insufficient in Part of the Institutions

**Figure 4:**
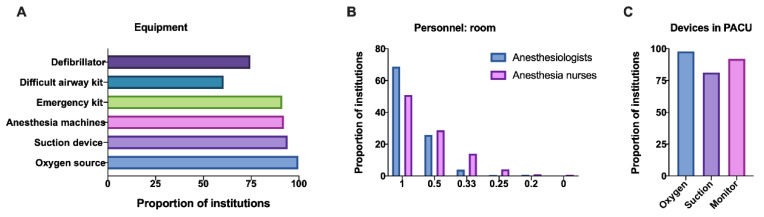
The facilities and personnel. Responses to these questions: (**A**) Which facilities were equipped in the area were hysteroscopies were performed? (**B**) How many anaesthesiologists/ nurse anesthetists were there in each operation room? (**C**) The PACUs were equipped with which devices? Abbreviations: PACU, postanesthesia care unit.

Moreover, we investigated the equipped emergency support devices and who performed sedation in places where hysteroscopy was carried out. The findings indicated that most institutions were equipped with oxygen sources (99.7%), suction devices (94.0%), anaesthesia machines (92.1%) and emergency kits (91.2%). Additionally, 74.7% of the institutions were equipped with a defibrillator, and 60.6% had a difficult airway kit (Figure 4A).

Responses revealed that all the personnel performing sedation during hysteroscopic procedures in mainland China were anaesthesiologists. Approximately 68.7% of the institutions had an anaesthesiologist-to-operating room (A-to-OR) ratio of 1, which means the presence of one anaesthesiologist in each operating room. Moreover, 25.9% of the institutions had an A-to-OR ratio of 0.5, and very few institutions had a ratio of <0.5 (5.4%). Similarly, 51.0% of the institutions had one operating room with one nurse anaesthetist, 28.7% had a nurse anaesthetist-to-OR ratio of 0.5 and 20.3% had a ratio of <0.5 (Figure 4B). Therefore, the shortage of devices and personnel is a national problem that needs to be solved.

### 3.5. The Management of Postanaesthesia Resuscitation Was Weak

The postanaesthesia resuscitation siutation was another issue to be considered, because most hysteroscopy procedures in China were completed in an operating room. Amazingly, 47.6% of the institutions still performed hysteroscopy without a PACU. Additionally, we investigated the devices equipped in the PACU and revealed that approximately 97.9% of the institutions had independent oxygen sources per patient, 81.2% had independent suction facilities per patient and 91.9% had independent monitors in PACU (Figure 4C). Lack of postanaesthesia care can increase the incidence of hypoxemia and neglect attention during the occurrence of postoperative pain.

### 3.6. The Hysteroscopic Diameter Was Larger and Need Cervical Dilation in China

**Figure 5:**
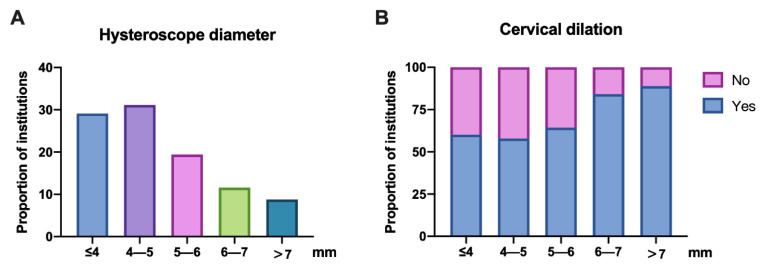
Hysteroscope lens diameter and situation of cervical dilatation. Responses to these questions: (**A**) What was the diameter of the hysteroscope lens used in your institution? (**B**) Did you dilate cervices before the hysteroscopic procedure?

The lens types and diameters used by institutions were surveyed to explore the factors that may influence patient discomfort during hysteroscopy. In terms of lens types, approximately 45.1% were rigid hysteroscopes, 4.5% were flexible hysteroscopes and 50.4% had both types (Supplementary Table S1). Hysteroscopic scissors, electrosurgery and both surgical methods were used by 18.2%, 17.2% and 64.6% of institutions, respectively, to treat uterine abnormalities, such as endometrial polyps, fibroids, septa and intrauterine adhesions (Supplementary Table S2). Additionally, the lens diameter of hysteroscopes varied in every institution, wherein 29.1% of institutions were ≤4 mm and 31.2% were between 4 and 5 mm. Approximately 39.7% of institutions used a larger instrument (>5 mm) (Figure 5A). Even in institutions using smaller-diameter hysteroscopes (≤4 mm), 60.2% of gynaecologists chose to dilate cervices before the hysteroscopic procedure. Additionally, the proportion of cervical dilatation increases as the lens diameter increases. Among the institutions with a lens diameter of 6–7 mm and >7 mm, 84.1% and 88.9% of gynaecologists, respectively, chose to require cervical dilation (Figure 5B).

### 3.7. Volume Overload Were the Most Common Complications during Hysteroscopy

This survey investigated the occurrence of four serious complications during the perioperative period of hysteroscopy in 2019–2021. Volume overload was the most common complication, occurring in 36.1% of hospitals, followed by uterine perforation (28.7%), pulmonary embolism (3.7%) and death within 24 h (1.1%) (Supplementary Table S3).

## 4. Discussion

The present survey described an overview of the status of hysteroscopy in China. This national survey revealed the large number of hysteroscopies performed in China every year and the high proportion of anaesthesia administration. Sedation was the most frequently used anaesthesia method, and propofol and opioid analgesics were the most popular agents. Additionally, our study suggested that all the sedation was performed by anaesthesiologists in China and the personnel was insufficient in most hospitals. The same problem existed with the equipment. Many institutions lacked emergency support devices, such as defibrillators and difficult airway kits. Even some institutions did not set up a PACU after sedation. The main factor causing patient discomfort may be the larger hysteroscope size and cervical dilation.

The overall number of hysteroscopies was >500,000 in 2021 according to our survey. The demand for assisted reproduction and the number of hysteroscopies will increase accordingly, with the opening of the three-child policy in China. The average percentage of anaesthesia was as high as 61.9%, which was different from other countries. The American College of Obstetricians and Gynaecologists and the American Association of Gynaecologic Laparoscopists (AAGL) recommended a single agent or a combination of multiple agents, including topical anaesthetics, NSAIDs, acetaminophen, benzodiazepine, opiate and intracervical or paracervical block, for pain management. However, no clinically significant difference was found in the safety or effectiveness of these regimens for pain management when compared to each other or placebo based on the currently available evidence [16]. Another guideline for hysteroscopy clinical practice from the French College of Gynaecologists and Obstetricians suggested that diagnostic hysteroscopies can be conducted without general anaesthesia (nor neuroleptanalgesia, nor conscious sedation), or regional anaesthesia. Additionally, regional anaesthesia and simple sedation were options for operative hysteroscopy [17]. The Polish Society of Gynecologists and Obstetricians Guidelines for the application of hysteroscopy recommend using a paracervical block with lidocaine or mepivacaine to reduce pain, especially in cases when cervical dilation is necessary or when intrauterine lesions are to be removed. However, this guideline does not recommend sedation due to the strict monitoring of vital signs and the associated potential complications [18]. The high proportion of anaesthesia in China might be due to cultural factors and different understandings of anaesthesia and surgery. Substantial numbers of patients in China believed that anaesthesia should induce sleep; hence, they urged deeper sedation due to the fear of feeling the operation process. Amongst various anaesthesia methods, sedation was a popular choice and the proportion of sedation was >75% in 44.2% of institutions. Moreover, anaesthesia was chosen mainly due to operative requirements and patient comfort. Therefore, sedation significantly decreased patient discomfort and anxiety and increase the success rates of surgery. Cornelissen and colleagues reported the safety and feasibility of therapeutic hysteroscopy under procedural sedation in an outpatient setting, with a high patient satisfaction rate [14]. A 2021 systematic review revealed that sedation significantly reduced intraprocedural pain when compared to local anaesthesia. This review emphasised that deeper sedation resulted in side effects, such as delirium, increased levels of postprocedural attention and increased staff requirements, although patients and hysteroscopists were more satisfied with deeper levels of sedation. Additionally, responsiveness was always suppressed as the level of sedation increased, and was the potential ability of the patient to maintain control of their airway, ventilation and cardiovascular function [19].

The type of hysteroscope and its lens diameter could be another factor influencing the high proportion of anaesthesia and analgesia use in China. Smaller hysteroscopes (≤4 mm) were applied in only 29.1% of institutions in China, while 39.7% used hysteroscopes with a diameter of >5 mm. The reduction in instrument size is crucial for minimizng pain and the risk of vasovagal reactions [10]. De Placido and colleagues revealed that the mean Visual Analogue Scale score for pain was significantly lower when using a 3.5 mm hysteroscope compared to a traditional 5 mm instrument [20]. Another randomised clinical trial (RCT) not only demonstrated a significantly lower mean level of pain experienced during minihysteroscopy compared to conventional instruments but the number of instrumentally recorded and clinical vasovagal reactions was also significantly lower in the minihysteroscopy group [21]. A systematic review of 10 RCTs revealed that smaller and mechanical devices, such as hysteroscopic tissue-retrieval systems and scissors, were associated with better pain control compared to larger or electrosurgical hysteroscopic instruments [22].

Additionally, the use of saline solution rather than carbon dioxide (CO_2_) as a distention medium, the vaginoscopic or traditional approach, the use of flexible instruments, and operator skill may have a role in pain intensity. Two types of lenses were used in hysteroscopy, including rigid and flexible hysteroscopes, which were used by 45.1% and 4.5% of institutions, respectively. Both types of hysteroscopes were applied in 50.4% of institutions in China. Flexible hysteroscopy is associated with less pain both at the introduction of the hysteroscope and during the procedure itself, especially when the diameter of the scope is reduced when compared with rigid hysteroscopy [23]. However, rigid hysteroscopes provide superior optical qualities and permit a more rapid performance with higher success rates at a much lower cost [24]. Operative hysteroscopy with a hysteroscope of >5 mm requires anaesthesia and cervical dilatation [25]. Our study found that most hysteroscopists would dilate the cervices before hysteroscope insertion, especially with larger hysteroscopic instruments, which might be a nonnegligible source of pain during the hysteroscopic procedures.

Some studies advocated office hysteroscopy, which could be feasibly performed in the office setting rather than in the operating room [26,27]. Office hysteroscopy was considered an efficient, cost-effective procedure, and could streamline care and avert anaesthesia exposure [14,28]. According to a survey by Helena, outpatient hysteroscopy was offered by 76.5% of United Kingdom gynaecologists [29]. However, only an estimated 15–25% of gynaecologists in the United States currently offer office hysteroscopy [30]. The situation is similar in China, and most hysteroscopic procedures are provided in the context of an institutional operating room setting, usually with the support of an anaesthesiologist providing some type of sedation, or general anaesthesia. Moreover, procedural sedation and analgesia can be provided by different kinds of healthcare professionals, such as anaesthesiologists, gastroenterologists, emergency medical physicians and nurses. However, all sedation for hysteroscopy was performed by anaesthesiologists in China mainland, which was different from other countries. This situation increased the demand for anaesthesiologists and brought the problem of personnel insufficiency. Our investigation revealed that only 68.7% of the institutions had one anaesthesiologist in each operating room, and 51.0% of the institutions had one operating room with one nurse anaesthetist in China. Sedation provided by anaesthesiologists significantly reduced sedation-related complications from 0.38% to 0.08% compared with nurse-administered sedation [31]. Buxbaum and colleagues compared the sedation effect of endoscopic retrograde cholangiopancreatography and endoscopic ultrasonography under the guidance of anaesthesiologists and gastroenterologists, respectively, and revealed higher quality and efficiency of anaesthesia under the guidance of anaesthetists [32]. Personnel insufficiency is a prevalent problem in every medical institution, and this problem is more prominent, especially when weighing safety and efficiency.

Our survey results indicated that the institutions in China monitored SPO_2_ (98.9%), ECG (91.6%) and NIBP (91.3%) most frequently during hysteroscopic procedures, but few hospitals monitored ETCO_2_ (38.0%). Practice guidelines for moderate procedural sedation and analgesia from the American Society of Anaesthesiologists suggested that adequate ventilation should be evaluated via continuous capnographic monitoring [33]. The lack of ETCO_2_ monitoring makes the anaesthesiologists unable to promptly recognise the occurrence of apnoea, especially in sedation, which can increase the risk of CO_2_ accumulation and hypercapnia, prolong the patient’s awake time, and increase the incidence of postoperative pulmonary complications [34,35]. Additionally, we noticed that only 74.7% of the units were equipped with defibrillators in the area where hysteroscopy was performed, and 60.6% of the units were equipped with difficult airway vehicles. Surprisingly, 47.6% of centres did not have a PACU. Inadequate rescue facilities made it impossible for patients to obtain first-aid measures in the shortest time when an emergency occurred, which greatly increased morbidity and mortality [36]. Moreover, HFNO is considered the best noninvasive choice for supplying oxygen, which is applied widely in patients with acute respiratory failure and coronavirus disease 2019 [37,38]. It can provide heated and humidified, titrated and regulated oxygen concentration via a nasal catheter. Our previous research revealed an advantage of HFNO in reducing the incidence of hypoxia during sedation for hysteroscopy [39]. However, only 1.1% of institutions used HFNO in the practice of sedation of hysteroscopic procedure in our investigation.

This study is a national survey of anaesthesia status for hysteroscopy performed in the medical institutions of assisted reproductive technology in mainland China. We revealed that anaesthesia was generally required during the hysteroscopic procedure, which was the biggest difference from other countries. To date, no convincing evidence can determine which regimen is the best for pain management in hysteroscopy, and additional large sample, multi-centre RCTs are warranted. This investigation revealed several problems existing in our routine anaesthesia of hysteroscopy, which motivates us to improve the safety and effectiveness of anaesthesia management.

Our study has limitations. Response bias might have affected our results because we did not know how representative our sample was for all anaesthesiologists and gynaecologists. Otherwise, the seniority of doctors was not included in our questionnaires, as different doctors may have different comprehension of the questions. However, we conducted telephone or letter follow-ups to ensure data accuracy in the step of data cleaning and checking. We did not investigate whether the hysteroscopy was performed in an office or operating room, which may influence the selection of instruments. Moreover, the level of sedation was not analysed in the survey, and conscious sedation was not distinguished. Sedation means deep sedation in our traditional clinical practice, in which patients are unconscious during the procedures.

## 5. Conclusions

Mainland China has large numbers of hysteroscopic procedures that are increasing yearly. The proportion of anaesthesia for hysteroscopy was approximately 63.8% in 2021. Sedation is currently prevalent in anaesthesia programmes. All the sedations were performed by anaesthesiologists. However, problems existed, such as a lack of first-aid devices, insufficient personnel and weak resuscitation management after anaesthesia. The lens diameter of hysteroscopes varied in every institution, and the mini-hysteroscope was applied in only 29.1% of institutions. 

## Figures and Tables

**Table 1:** Responses to the question: What was the proportion of anesthesia applied in hysteroscopic procedure in your institution in 2021?

Proportion of Anesthesia	0–25%	25–50%	50–75%	75–100%
Sedation	116 (32.7%)	40 (11.3%)	42 (11.8%)	157 (44.2%)
General anesthesia with LMA	252 (71.0%)	38 (10.7%)	19 (5.3%)	46 (13.0%)
General anesthesia with ETT	300 (84.5%)	29 (8.1%)	13 (3.7%)	13 (3.7%)
Local anesthesia	293 (82.5%)	37 (10.4%)	12 (3.4%)	13 (3.7%)
Intraspinal anesthesia	308 (86.8%)	29 (8.2%)	12 (3.4%)	6 (1.6%)

Details were showed as number of institutions (%). Abbreviations: LMA, laryngeal mask airway; ETT, endotracheal tube.

## Data Availability

All relevant data and study protocols are shown in the manuscript. All requests for or questions about the data can be available from the corresponding author on reasonable request.

## Supplementary Materials

The following supporting information can be downloaded at: https://www.mdpi.com/article/10.3390/jpm13101436/s1, Table S1: Responses to the question: What was the type of hysteroscope lens used in your institution? Table S2: Responses to the question: What was the method used by your institution for operative hysteroscopy (To treat the uterine abnormalities such as endometrial polyps, fibroids, septa, and intrauterine adhesions)? Table S3: Responses to the question: which of the severe complications occurred in your institution in 2021? Material S1: Questionnaire of hysteroscopy for anesthesiologists; Material S2: Questionnaire of hysteroscopy for gynaecologists.
