# Supplementary material for: The Current Situation of Anaesthesia for Hysteroscopy in Mainland China: A National Survey
Source: J Pers Med. 2023 Sep 26;13(10):1436. doi: 10.3390/jpm13101436 (PMC10608545; doi:10.3390/jpm13101436)
Supplement: Supplementary file 1 [file jpm-13-01436-s001.zip › Supplementary Material S1 Questionnaire for anesthesiologists.pdf]

Supplementary Material S1

Questionnaire of hysteroscopy for anesthesiologists

Dear Sir/Madam:

Hysteroscopy can directly observe and deal with intrauterine abnormalities, which plays an important role in assisted reproductive technology. However, the selection of lens type, operation and anesthesia method varied in each assisted reproduction institutions. Therefore, we designed this questionnaire to investigate the current status of hysteroscopy in each institution, and comprehensively understand the factors that influence the selection of anesthesia methods, so as to improve the safety and effectiveness of anesthesia management. Please take a few minutes to fill in the questionnaire carefully.

This is a voluntary survey. Before starting to answer this questionnaire, we want to collect your information to validate data, including your surname, institution name, and telephone number. And we guarantee that your responses and privacy would be maintained in password-protected computers, any identifying information would be delinked after analysis and publication. The questions can be answered only after you agree with these items. Please make sure that the numbers you fill in are accurate and reliable. Thank you for your cooperation!

Yours, sincerely
Diansan Su
May 2022

Q1. Your surname: ____
Your institution name: ____
Telephone number: ____

Q2. Does your reproductive center carry out hysteroscopy? And in which year did it carry out the first case? (If the answer is “yes”, please going to Q2; otherwise, ending)

☐ Yes, in ____year ☐ No

Q3. What was the proportion of anesthesia applied in hysteroscopic procedure in your reproductive institution in 2021? (If the selected percentage was more than 50%, please go to Q4; otherwise, go to Q5 directly)

☐ 0-25%, the actual percentage was ____
☐ 25-50%, the actual percentage was ____
☐ 50-75%, the actual percentage was ____
☐ 75-100%, the actual percentage was ____

Q4. What were the reasons for high proportion of anesthesia in your institution? (Multiple choice)

☐ operation requirements
☐ to make patient comfortable

☐ patient's strong will

Q5. What was the proportion of intravenous sedation for hysteroscopy in 2021?
☐ 0-25%, the actual percentage was ____
☐ 25-50%, the actual percentage was ____
☐ 50-75%, the actual percentage was ____
☐ 75-100%, the actual percentage was ____

Q6. What was the proportion of general anesthesia with endotracheal tube intubation for hysteroscopy in 2021?
☐ 0-25%, the actual percentage was ____
☐ 25-50%, the actual percentage was ____
☐ 50-75%, the actual percentage was ____
☐ 75-100%, the actual percentage was ____

Q7. What was the proportion of general anesthesia with laryngeal mask airway for hysteroscopy in 2021?
☐ 0-25%, the actual percentage was ____
☐ 25-50%, the actual percentage was ____
☐ 50-75%, the actual percentage was ____
☐ 75-100%, the actual percentage was ____

Q8. What was the proportion of intraspinal anesthesia for hysteroscopy in 2021?
☐ 0-25%, the actual percentage was ____
☐ 25-50%, the actual percentage was ____
☐ 50-75%, the actual percentage was ____
☐ 75-100%, the actual percentage was ____

Q9. What was the proportion of local anesthesia for hysteroscopy in 2021 (Including paracervical block; intracervical anaesthesia; topical anaesthesia, e.g. sprays and gels; intrauterine anaesthesia, et al.)?
☐ 0-25%, the actual percentage was ____
☐ 25-50%, the actual percentage was ____
☐ 50-75%, the actual percentage was ____
☐ 75-100%, the actual percentage was ____

Q10. Who performed the intravenous sedation in your institution?
☐ Anesthesiologists □ Nurse anesthetists □ Gynaecologists □ Others

Q11. What was the most common used sedatives of intravenous sedation during hysteroscopy?
□ Propofol □ Etomidate □ Midazolam □ Remimazolam

Q12. What was the most common used analgesics of intravenous sedation during hysteroscopy?
□ Fentanyl □ Sufentanil □ Remifentanil □ Alfentanil □ Dezocine

□ Butorphanol □ Nalbuphine □No analgesics

Q13. Which oxygen delivery technique was applied in intravenous sedation of hysteroscopy?
□ Regular nasal catheter oxygen (1~6 L/min)
□ Standard mask
□ High concentration mask
□ High flow nasal oxygen (>30L/min)
□ Others, ____
□ None

Q14. Which monitoring parameters were used during intravenous sedation of hysteroscopy?
□ Pulse oxygen saturation (SPO2)
□ Electrocardiography (ECG)
□ Non-invasive blood pressure (NIBP)
□ Respiratory rate (RR)
□ End-tidal carbon dioxide ($ETCO_2$)
□ Bispectral index (BIS)
□ Others, ____

Q15. Which facilities were equipped in the area that hysteroscopies were performed? (Multiple choice)
□ Oxygen sources
□ Suction devices
□ Anesthesia machines
□ Emergency kit
□ Difficult airway kit
□ Defibrillator

Q16. How many serious complications occurred in your institution between 2019 and 2021? (Multiple choice)
☐ Volume overload, ____ cases
☐ Uterine perforation, ____ cases
□ Death within 24 hours, ____ cases
□ Pulmonary embolism, ____ cases

Q17. How many anaesthesiologists were there in each operation room?
☐ 1 □ 2 □ 3 □ 4 □ 5

Q18. How many nurse anesthetists were there in each operation room?
□ 1 □ 2 □ 3 □ 4 □ 5

Q19. Was there a post-anesthesia care unit in the hysteroscopy operating room of your institution? (If the answer is “yes”, please going to Q20; otherwise, ending)
☐ Yes □ No

Q20. Which of the following devices were equipped in PACU?

□ Independent oxygen source for each patient

□ Independent suction for each patient

□ Independent monitor for each patient
