# Supplementary material for: The Current Situation of Anaesthesia for Hysteroscopy in Mainland China: A National Survey
Source: J Pers Med. 2023 Sep 26;13(10):1436. doi: 10.3390/jpm13101436 (PMC10608545; doi:10.3390/jpm13101436)
Supplement: Supplementary file 1 [file jpm-13-01436-s001.zip › Supplementary Material S2 Questionnaire for gynaecologists.pdf]

Supplementary Material S2

Questionnaire of hysteroscopy for gynaecologists

Dear Sir/Madam:

Hysteroscopy can directly observe and deal with intrauterine abnormalities, which plays an important role in assisted reproductive technology. However, the selection of lens type, operation and anesthesia method varied in each assisted reproduction institutions. Therefore, we designed this questionnaire to investigate the current status of hysteroscopy in each institution, and comprehensively understand the factors that influence the selection of anesthesia methods, so as to improve the safety and effectiveness of anesthesia management. Please take a few minutes to fill in the questionnaire carefully.

This is a voluntary survey. Before starting to answer this questionnaire, we want to collect your information to validate data, including your surname, institution name, and telephone number. And we guarantee that your responses and privacy would be maintained in password-protected computers, any identifying information would be delinked after analysis and publication. The questions can be answered only after you agree with these items. Please make sure that the numbers you fill in are accurate and reliable. Thank you for your cooperation!

Yours, sincerely
Diansan Su
May 2022

Q1. Your surname: ____
Your institution name: ____
Telephone number: ____

Q2. Does your reproductive center carry out hysteroscopy? And in which year did it carry out? (If the answer is “yes”, please going to Q3; otherwise, ending)
□ Yes, in ____year □ No

Q3. How many hysteroscopies were carried out in your institution in 2019?
□ <2,000, ____cases □ 2,000-4,000, ____cases □ 4,000-6,000, ____cases
□ 6,000-8,000, ____cases □ 8,000-10,000, ____cases □ 10,000-12,000, ____cases
□ >12,0000, ____cases

Q4. How many hysteroscopies were carried out in your institution in 2020?
□ <2,000, ____cases □ 2,000-4,000, ____cases □ 4,000-6,000, ____cases
□ 6,000-8,000, ____cases □ 8,000-10,000, ____cases □ 10,000-12,000, ____cases
□ >12,0000, ____cases

Q5. How many hysteroscopies were carried out in your institution in 2021?
□ <2,000, ____cases □ 2,000-4,000, ____cases □ 4,000-6,000, ____cases
□ 6,000-8,000, ____cases □ 8,000-10,000, ____cases □ 10,000-12,000, ____cases

□ >12,0000, ____cases

Q6. What was the type of hysteroscope lens used in your insitution?

☐ Rigid hysteroscope □ Flexible hysteroscope □ Both

Q7. What was the method used by your institution for operative hysteroscopy (To treat the uterine abnormalities such as endometrial polyps, fibroids, septa, and intrauterine adhesions)?

☐ Hysteroscopic scissors □ Electrosurgery □ Both

Q8. What was the diameter of the hysteroscope lens in your institution?

□ ≤4 mm □ 4-5 mm □ 5-6 mm □ 6-7 mm □ >7 mm

Q9. Did you dilate cervices before the hysteroscopic procedure?

□ Yes □ No
