# Supplementary material for: The Current Situation of Anaesthesia for Hysteroscopy in Mainland China: A National Survey
Source: J Pers Med. 2023 Sep 26;13(10):1436. doi: 10.3390/jpm13101436 (PMC10608545; doi:10.3390/jpm13101436)
Supplement: Supplementary file 1 [file jpm-13-01436-s001.zip › Supplementary Tables.pdf]

Supplementary Table S1. Responses to the question: What was the type of hysteroscope lens used in your institution?

Types	Number of institutions (%)
Flexible hysteroscopes	12 (4.5%)
Rigid hysteroscopes	121 (45.1%)
Both	135 (50.4%)

Supplementary Table S2. Responses to the question: What was the method used by your institution for operative hysteroscopy (To treat the uterine abnormalities such as endometrial polyps, fibroids, septa, and intrauterine adhesions)?

Methods	Number of institutions (%)
Hysteroscopic scissors	49 (18.2%)
Electrosurgery	46 (17.2%)
Both	173 (64.6%)

Supplementary Table S3. Responses to the question: which of the severe complications occurred in your institution in 2021?

Complications	Number of institutions (%)
Volume overload	128 (36.1%)
Uterine perforation	101 (28.5%)
Pulmonary embolism	13 (3.7%)
Death within 24 hours	4 (1.1%)
